# Efficient Induction of Apoptosis by Wee1 Kinase Inhibition in Hepatocellular Carcinoma Cells

**DOI:** 10.1371/journal.pone.0100495

**Published:** 2014-06-24

**Authors:** Tomomi Kogiso, Hikaru Nagahara, Etsuko Hashimoto, Shunichi Ariizumi, Masakazu Yamamoto, Keiko Shiratori

**Affiliations:** 1 Institute of Gastroenterology, Department of Internal Medicine, Tokyo Women's Medical University, Tokyo, Japan; 2 Aoyama hospital, Tokyo Women's Medical University, Tokyo, Japan; 3 Institute of Gastroenterology, Department of Surgery, Tokyo Women's Medical University, Tokyo, Japan; The University of Tokyo, Japan

## Abstract

Transforming growth factor-β1 (TGF-β1) potently inhibits human hepatocellular carcinoma (HCC) cell growth. Here we demonstrated that TGF-β1-induced apoptosis is mediated by decreased phosphorylation of cdc2 at Tyr15 accompanied by down-regulation of Wee1 kinase expression. As expected from these results, a Wee1 kinase inhibitor efficiently induced apoptosis in HCC cells in the absence of TGF-β1 treatment. In surgically resected samples, Wee1 kinase was expressed in moderately to poorly differentiated HCC, whereas no Wee1 kinase expression was observed in non-cancerous tissue, including cirrhotic tissue. Our results suggest that Wee1 kinase inhibitors may be a practical novel therapeutic option against advanced HCC.

## Introduction

Human hepatocellular carcinoma (HCC) is a widespread disease brought about by long-term liver inflammation [1], [2]. Although surgical and percutaneous radiofrequency ablation improve patient survival, new therapeutic options are needed to achieve effective treatment of advanced HCC [3]–[6]. Apoptotic induction by chemotherapy and low molecular weight material delivery are both promising options [6], [7].

Previous studies showed that transforming growth factor-β1 (TGF-β1) stimulates cell proliferation in non-epithelial cells, such as fibroblasts and stellate cells, whereas it suppresses cell growth in both rodent and human HCC cells by inducing apoptosis or cell cycle arrest [8], [9]. However, the main molecules involved in TGF-β1-induced apoptosis in HCC cells are largely unknown.

In this study, we evaluated human HCC cell lines to elucidate TGF-β1-induced apoptotic mechanisms. Our results demonstrated that we efficiently induced apoptosis in HCC cell lines using a Wee1 kinase inhibitor. These results may lead to development of novel therapeutic options against human HCC.

## Materials and Methods

### Cell culture

The human hepatocellular carcinoma cell line, HuH7, was purchased from the Riken Cell Bank (Wako, Saitama, Japan). The cells were maintained in Dulbecco's Modified Eagle Medium (DMEM) supplemented with 5% heat-inactivated fetal calf serum (FCS). TGF-β1-induced apoptosis-resistant HuH7 cells (HuH7R) were established from the HuH7 line by maintaining the cells with low-dose TGF-β1 (0.2 µg/mL; R&D Systems, Minneapolis, MN, USA) for 1-2 months. TGF-β1-induced apoptotic resistance was confirmed by flow cytometry following TGF-β1 treatment.

### Flow cytometric analyses

Cells were trypsinized, harvested, and fixed with 70% ethanol at −20°C for 1 h. After washing with phosphate buffered saline (PBS), the cells were stained with propidium iodide (PI), and the cell cycle was analyzed using a FACScan (Becton Dickinson, Franklin Lakes, NJ, USA).

### Cell treatment

For TGF-β1 treatment, cells were incubated in medium containing 0.1% FCS and 2 µg/mL TGF-β1. To counteract TGF-β1-mediated apoptosis, 20 µM roscovitine (Calbiochem, Nottingham, UK) was added to the medium 1 h prior to TGF-β1.

PD166285, a Wee1 kinase inhibitor, was provided by Pfizer (Ann Arbor, MI, USA) and used at a concentration of 200 nM. Roscovitine (20 µM) was also added 1 h prior to PD166285 administration to inhibit cdc2 activity resulting from the inhibition of PD166285-mediated apoptosis.

### Immunoprecipitation and immunoblotting

Cells were lysed using 0.4 mL E1A lysis buffer [ELB: 50 mM HEPES (pH 7.2) 250 mM NaCl, 2 mM EDTA, 0.1% Nonidet P-40, 1 mM DTT, 1 µg/mL aprotinin, 1 µg/mL leupeptin, 50 µg/mL phenylmethylsulfonyl fluoride, 0.5 mM NaP_2_O_7_, 0.1 mM NaVO_4_, and 5.0 mM NaF] (all reagents were purchased from Sigma-Aldrich, St Louis, MO, USA). The lysed cell solution was centrifuged, protein G or cdc2 antibodies were added to the supernatant, and the mixture was incubated at 4°C overnight. Immunoblots were prepared as described previously [10] and probed with anti-Wee1, anti-β-actin (Santa Cruz Biotechnology, Santa Cruz, CA, USA), or anti-cdc2-phospho-Tyr15 (Cell Signaling Technology, Danvers, MA, USA).

### Kinase assay

TGF-β1-treated or untreated whole cell extracts were incubated with an anti-cdc2 antibody overnight at 4°C. The precipitates were washed and incubated with α-^32^P-ATP for 20 min at 30°C in a kinase solution [10] containing histone H1 (Sigma-Aldrich), as a substrate of cdc2, and then subjected to sodium dodecyl sulfate polyacrylamide gel electrophoresis (SDS-PAGE). The incorporation rate of ^32^P into histone H1 was measured using a PhosphoImager (BioRad Laboratories Hercules, CA, USA).

### Transfection of short interfering RNA (siRNA)

siRNAs against Wee1 kinase were synthesized and cloned into a piGENEPURhU6 vector (Toyobo, Tokyo, Japan) and named psiWee1. psiWee1 was transfected into HuH7 cells using Optifect Reagent (Invitrogen, Carlsbad, CA, USA). The target sequences against Wee1 kinase were GGCAGAAGATGATCATATG (18-2) and GGCAGAAGCTGATCTTCTC (18-3).

### Immunohistostaining

HCC tissue samples (n = 26) were obtained by surgical resection and authorized for immunohistochemical analysis after receiving written informed consent from each patient. This study was approved by the ethics committee of Tokyo Women's Medical University Hospital (Tokyo, Japan). The tissue sections were placed into 10 mM EDTA (pH 9.0), heated at 90–95°C for 40 minutes, and then incubated with normal rabbit serum and reacted with an anti-Wee1 antibody (1∶200; Santa Cruz Biotechnology). We used DAKO Envision+System (DAKO, Glostrup, Denmark) horseradish peroxidase (HRP) as a secondary antibody according to the manufacturer's instructions and detected signals using 3,3′ diaminobenzidine (DAB) as a substrate.

### Statistical analyses

Significant differences between groups were determined using a Student's *t*-test or a chi-square test. P<0.05 was considered to be statistically significant.

## Results

### TGF-β1 induces apoptosis in HCC cells by cdc2 activation

**Figure 1:**
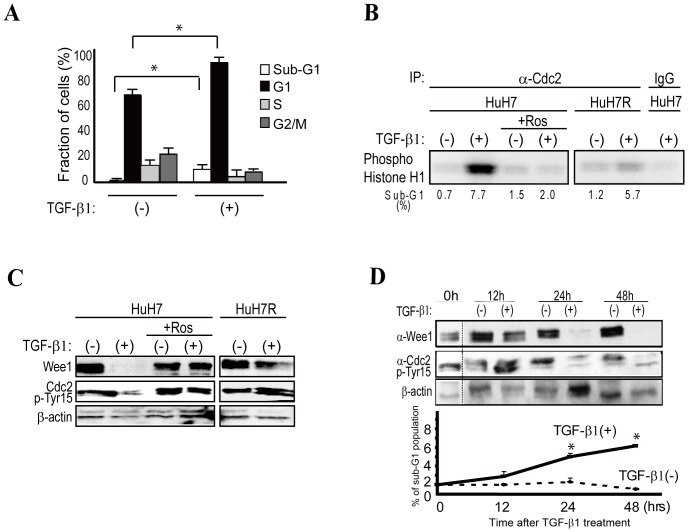
TGF-B1 induces apoptosis through cdc2 activation. **A,** FACS analyses showed that the sub-G1 phase HuH7 cell population increased from approximately 0.7% to 7.7%, and cells in the G1 phase increased from approximately 69% to 87% 48 h after TGF-β1 treatment. Data represent the means ± S.D. of three experiments. * indicates significant differences between each group (P<0.05). B, Cdc2 kinase activity was determined based on the level of phosphorylated histone H1 using histone H1 as a substrate. Cdc2 was activated 48 h after TGF-β1 treatment in HuH7 cells. However, cdc2 was not activated in HuH7R cells, which were isolated as an apoptosis-resistant clone from TGF-β1-treated HuH7 cells. Roscovitine (Ros)-pretreated HuH7 cells did not show cdc2 activation. A representative image from three independent experiments is shown. **C,** After TGF-β1 treatment (48 h), we observed cdc2 Tyr15 dephosphorylation in association with Wee1 kinase down-regulation in apoptotic cells. Pretreatment with 20 µM roscovitine completely abolished apoptosis and restored Wee1 kinase expression. TGF-β1 treatment induced G1 cell cycle arrest in HuH7R cells; however, Wee1 kinase expression and non-phosphorylated cdc2 Tyr 15 were similar to those of roscovitine-pretreated HuH7 cells. A representative image of three experiments is shown. **D,** Wee1 down-regulation and cdc2 Tyr15 dephosphorylation commenced approximately 24 h after TGF-β1 treatment, which was similar to thea initiation of apoptosis. A representative Western blot image is shown in the upper panels. The results in the lower graphs represent the means ± S.D. of three experiments. * indicates significant differences between each group (P<0.05).

TGF-β1 administration resulted in an increased proportion of the HuH7 cell population in the sub-G1 (demonstrative of apoptosis) and G1 phases (demonstrative of cell cycle arrest) (Figure 1A). FACS analyses revealed that the sub-G1 phase cell population increased from approximately 0.7% to 7.7%, and HuH7 cells in the G1 phase increased from approximately 69% to 87% after 48 h of TGF-β1 treatment (Figure 1A and B).

Consistent with previous reports [11], cdc2 was activated after TGF-β1 treatment in HuH7 cells (Figure 1B). We then examined whether cdc2 activity was necessary for the induction of apoptosis. We established an apoptosis-resistant HuH7 clone (HuH7R) that we isolated after long-term culture under low TGF-β1 concentration conditions. In these HuH7R cells, cdc2 was not activated, even after TGF-β1 treatment (Figure 1B). To further validate the role of cdc2 activation in the induction of apoptosis after TGF-β1 stimulation, we pre-treated HuH7 cells with roscovitine, a chemical inhibitor of cdc2. Roscovitine pretreatment markedly decreased the sub-G1 cell population and the cdc2 activity even after TGF-β1 treatment (Figure 1B). These results suggest that cdc2 activation is important for TGF-β1-induced apoptosis.

Cdc2 activity is regulated by its phosphorylation at Tyr15 [12]–[16]; thus, we examined Wee1 kinase expression and cdc2 Tyr15 phosphorylation after TGF-β1 treatment. As predicted, Wee1 kinase expression was down-regulated, and cdc2 Tyr15 was dephosphorylated in TGF-β1-treated cells (Figure 1C). Apoptosis-resistant HuH7R cells also preserved Wee1 kinase expression and Tyr15 phosphorylation of cdc2 even after TGF-β1 stimulation (Figure 1C). In a time course experiment, down-regulation of Wee1 expression and Tyr15 dephosphorylation of cdc2 commenced approximately 24 h after TGF-β1 treatment and was consistent with the initiation of apoptosis (Figure 1D).

### Targeting Wee1 kinase induces apoptosis in HCC cells

**Figure 2:**
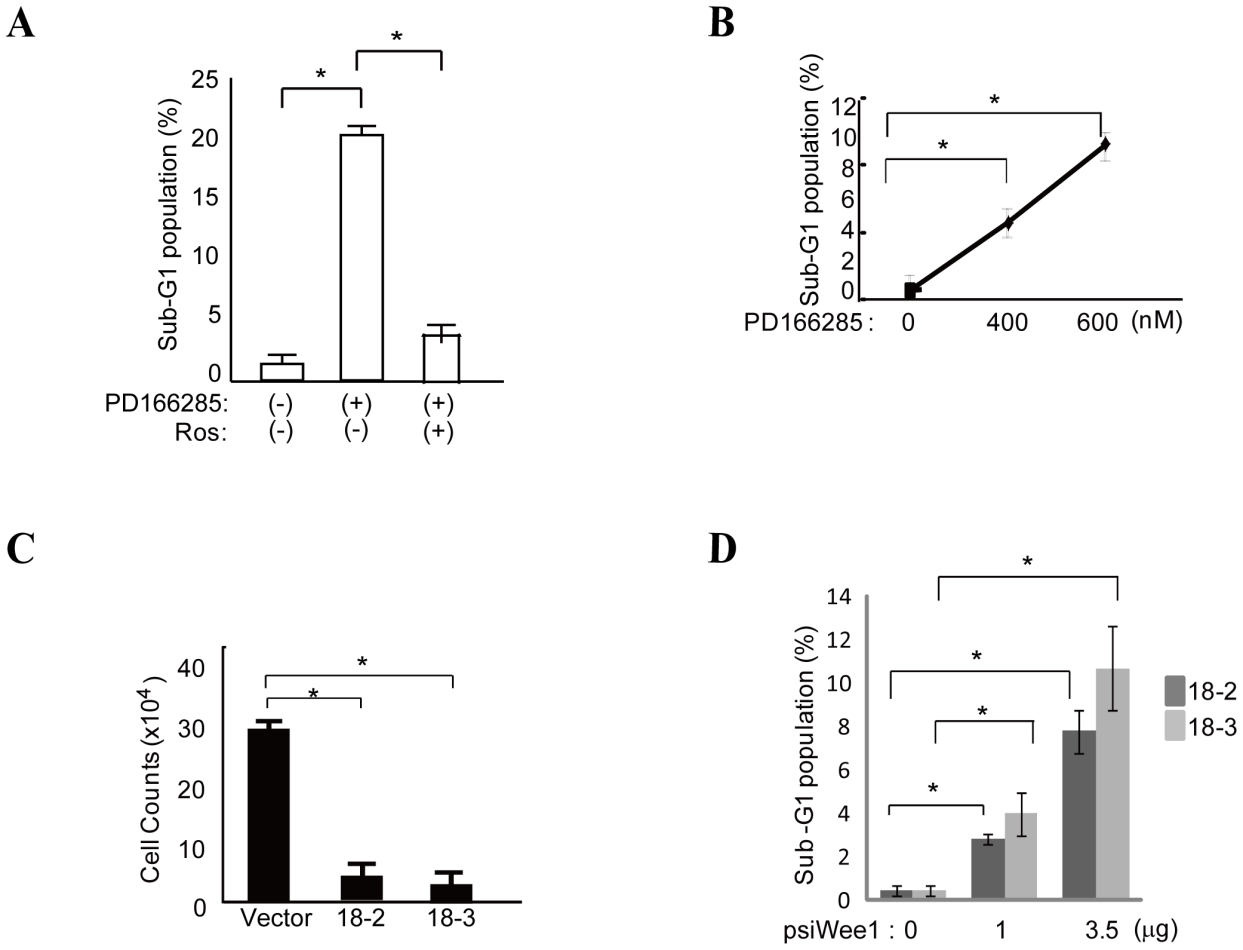
Targeting Wee1 kinase induces apoptosis in HCC cells. A, The number of sub-G1 HuH7 cells significantly increased after PD166285 treatment (200 nM). PD166285-mediated apoptosis of HuH7 cells was completely inhibited by pretreatment with 20 µM roscovitine (Ros). * indicates significant differences between each group (P<0.05). The results are presented as means ± S.D. of three experiments. **B,** PD166285 decreased the number of HuH7 cells in a concentration-dependent manner after 72 h. * indicates significant differences between each group (P<0.05). The representative results of three independent experiments are shown. **C** and D, Wee1 kinase-specific siRNAs (18-2 or 18-3) significantly reduced the total number of cells and increased the sub-G1 cell population 48 h after transfection in a concentration-dependent manner compared with control-vector transfectants. * indicates significant differences between each group (P<0.05).

Given the above findings, we tested whether apoptosis could be induced in HCC cells by inhibiting Wee1 kinase using a specific Wee1 kinase inhibitor, PD166285 [17], [18], or siRNA against Wee1 kinase in the absence of TGF-β1 stimulation. Treatment with PD166285 alone increased the sub-G1 population of HuH7 cells in a concentration-dependent manner (Figure 2A and B).

Next we investigated the effect of roscovitine treatment on PD166285-mediated apoptosis, since roscovitine can inhibit TGF-β1-mediated apoptosis (Figure 1B). As expected, roscovitine exclusively inhibited apoptosis mediated by the Wee1 kinase inhibitor (Figure 2A), suggesting that this apoptotic pathway is mediated by an active form of cdc2. Although these are possible molecular mechanisms for the induction of apoptosis by the Wee1 kinase inhibitor, we do not fully exclude other possibilities.

We subsequently used two different siRNAs (18-2 and 18-3) targeting separate sites of Wee1 kinase mRNA to inhibit Wee1 kinase expression. HuH7 cells were transfected with these Wee1 siRNAs, and the resulting cell number and sub-G1 population of the transfectants were analyzed (Figure 2C and D). Both siRNAs induced apoptosis efficiently, compared with vector alone (Figure 2C), in a concentration-dependent manner (Figure 2D).

### Wee1 kinase is expressed in moderately to poorly differentiated HCC but not in non-cancerous lesions

**Figure 3:**
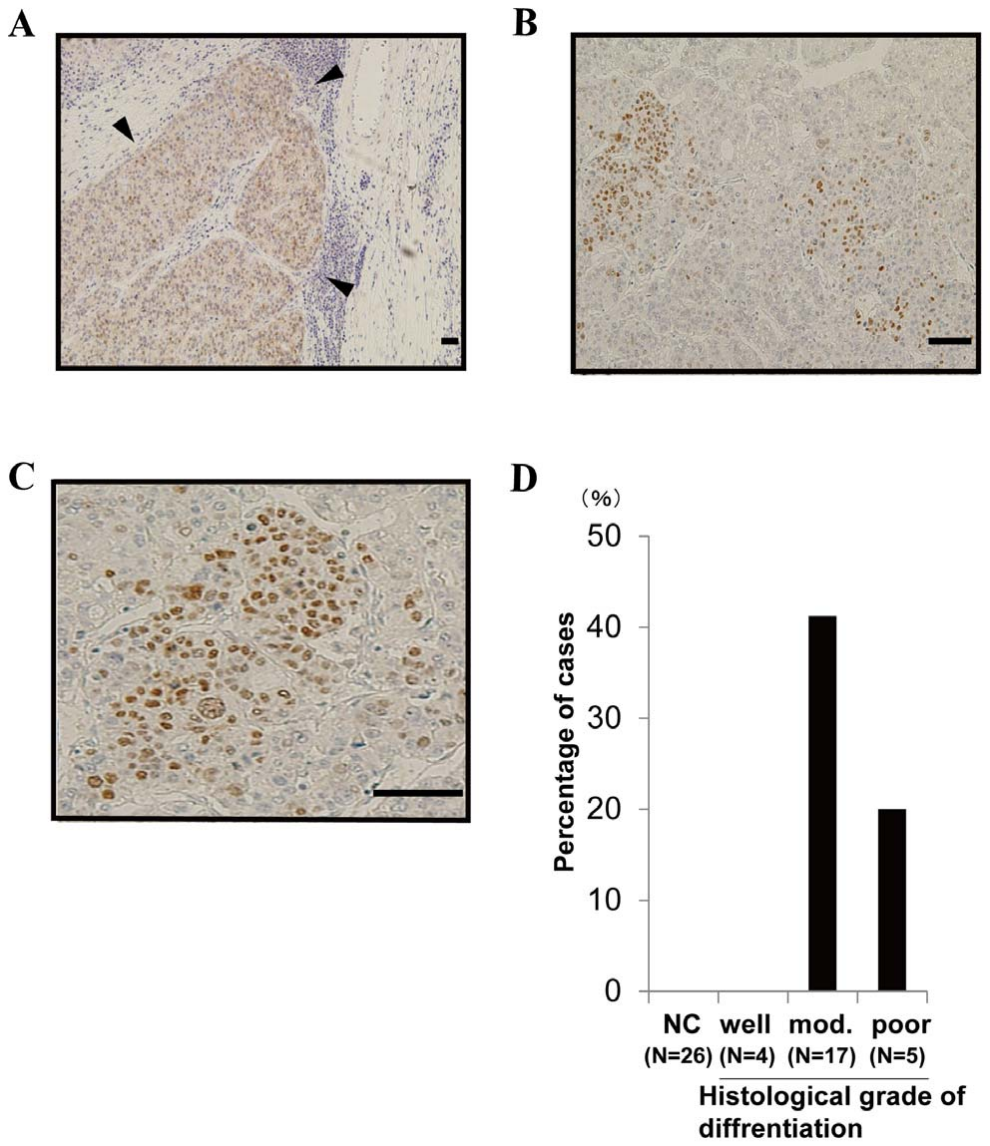
Wee1 kinase is expressed in moderately to poorly differentiated HCC. **A-C,** Wee1 kinase was stained with anti-Wee1 antibodies in surgically resected HCC samples (▾ HCC lesion). Anti-Wee1 antibody staining revealed mostly nuclear localization in Wee1 kinase-positive cells. The magnification is 40 x (A), 100 x (B), or 200 x (C). Scale bars  = 200 µm. **D,** Wee1 kinase is expressed in moderately to poorly differentiated HCC. A chi-square test revealed significant differences (p = 0.0026). (NC; Non-cancerous lesion, Mod.; Moderately).

To determine the expression of Wee1 kinase in human HCC, we obtained tissue samples from surgically resected HCC and performed immunohistochemistry. Wee1 kinase immunohistochemistry revealed positive nuclear staining in cells located at the margins of HCC and non-cancerous lesions (Figure 3A–C). Wee1 kinase expression was detected in 7/17 (41.2%) moderately differentiated HCC tissues and 1/5 (20%) poorly differentiated HCC tissues, while there were no apparent positive cells in four well-differentiated HCC tissues and the corresponding 26 non-cancerous lesions (Figure 3D, p = 0.0026).

## Discussion

TGF-β potently inhibits HCC cell growth by inducing cell cycle arrest and/or apoptosis [19]–[22]. Here we showed that the induction of apoptosis by TGF-β1 in HCC cells is mediated by de-phosphorylation of cdc2 Tyr15 (i.e., the active form of cdc2). This cdc2 activation was induced by Wee1 kinase down-regulation. In addition, we induced apoptosis in HCC cells by inhibiting Wee1 kinase using a specific inhibitor or siRNA.

Previous studies have identified several molecules that may play key roles in the induction or inhibition of apoptosis by TGF-β1 [23], [24]. We determined here that cdc2 activation due to decreased Tyr15 phosphorylation may be crucial for TGF-β1-induced apoptosis in HCC cells. We observed decreased Wee1 kinase expression after TGF-β1 treatment, and cdc2 phosphorylation is regulated by Wee1 kinase. Therefore, it is possible that TGF-β1-mediated apoptosis was induced by a Wee1/cdc2 axis. However, the molecular mechanisms underlying the TGF-β1-mediated downregulation of Wee1 kinase are unclear.

In surgically resected samples, Wee1 kinase was over-expressed in HCC, particularly in moderately to poorly differentiated or advanced HCC. Wee1 kinase is a negative regulator of cdc2; thus, questions remain regarding the role of increased Wee1 kinase in HCC.

Based on our results, we focused on Wee1 kinase as a possible therapeutic target. As expected, a kinase inhibitor and siRNAs against Wee1 kinase efficiently induced apoptosis in HCC cells in the absence of TGF-β1. Because TGF-β1 is a multifunctional cytokine, it may not be a practical therapeutic option.

Overexpression of Wee1 kinase has been reported in other tumor types, including brain tumors and leukemia, and the usefulness of Wee1 kinase inhibitor has been demonstrated [25], [26]. In brain tumors, the expression of Wee1 kinase was upregulated only in tumor cells, not in normal cerebellum, and the upregulated kinase had an important role in cancer cell survival [25]. Wee1 kinase negatively regulates G2/M phase by inhibiting the initiation of mitosis before DNA damage is repaired [27]–[29]. Thus, it is thought that Wee1 kinase abrogates premature mitotic entry and subsequent cell death [28], [29]. Therefore, the inhibition of Wee1 kinase may be a promising new therapeutic strategy in Wee1 kinase-overexpressing carcinomas [25].

Wee1 kinase was expressed focally in the carcinoma tissues. Thus, for clinical applications, there would be some limitations on effectiveness. However, a Phase II trial using a Wee1 kinase inhibitor for the treatment of advanced solid cancers is currently underway [29], [30]; thus, the use of this type of inhibitor may be a realistic therapeutic option against advanced HCC.
